# Rapid On-Site Detection of the *Bursaphelenchus xylophilus* Using Recombinase Polymerase Amplification Combined With Lateral Flow Dipstick That Eliminates Interference From Primer-Dependent Artifacts

**DOI:** 10.3389/fpls.2022.856109

**Published:** 2022-03-18

**Authors:** Qinzheng Zhou, Ya Liu, Zheng Wang, Huimin Wang, Xingyao Zhang, Quan Lu

**Affiliations:** Key Laboratory of Forest Protection of National Forestry and Grassland Administration, Research Institute of Forest Ecology, Environment and Nature Conservation, Chinese Academy of Forestry, Beijing, China

**Keywords:** pine wilt disease, rapid diagnostic, recombinase polymerase amplification, lateral flow dipstick, *Bursaphelenchus xylophilus*, POCT, false positive

## Abstract

The pine wood nematode (PWN), *Bursaphelenchus xylophilus*, is one of the most lethal nematode species, which causes pine wilt disease (PWD), a devastating forest disease. To date, no effective methods have been developed to control the disease; hence, rapid precise detection of *B. xylophilus* is of great significance. Traditional molecular diagnostic methods are time-consuming and require sophisticated instruments or skilled operators, which are unavailable in resource-limited settings. A specific, sensitive, and field-applicable diagnostic method is urgently needed. In this study, we developed a diagnostic method using recombinase polymerase amplification combined with lateral flow dipstick (RPA-LFD) for the rapid on-site detection of *B. xylophilus.* The false-positive signals from primer-dependent artifacts were eliminated using a probe, and base substitutions were included in the primer and probe. The entire detection process for the RPA-LFD assay can be completed under 38°C within approximately 30 min, including 15 min for crude nematode genomic DNA (gDNA) extraction and master mix preparation, 15 min for the RPA-LFD assay. This assay displayed high specificity toward *B. xylophilus* and showed no cross-reactions with closely related species, including *Bursaphelenchus mucronatus* and *Bursaphelenchus doui*. The sensitivity of this assay had a detection limit as low as 1 pg of *B. xylophilus* purified genomic DNA. Furthermore, the application of the RPA-LFD assay in simulated spiked pinewood samples showed accurate detection results. The RPA-LFD assay in this study successfully detected *B. xylophilus* in less than 30 min, providing a novel alternative for the simple, sensitive, and specific detection of *B. xylophilus* and showed potential for *B. xylophilus* point-of-care testing (POCT) in resource-limited areas or in field.

## Introduction

Pine wilt disease (PWD) is a devastating forest disease caused by the pine wood nematode (PWN) (Mota and Vieira, 2008). The spread of PWNs is mainly caused by increased global trade. As PWD is regarded as an “incurable disease,” rapid and accurate detection, quarantine, and monitoring can manage disease spread (Futai, 2013; Yazaki et al., 2018; Kim et al., 2020). Therefore, the primary task in PWD control is to accurately detect PWN.

Various *Bursaphelenchus xylophilus* diagnostic methods have been developed, including morphological methods using microscopic observations and molecular diagnostic methods using diverse molecular markers (Inácio et al., 2015; Lee et al., 2021). However, due to the similar morphological characteristics of *B. xylophilus* and its closely related species, *Bursaphelenchus mucronatus*, accurately identifying *B. xylophilus* requires specialized knowledge and professional skills (Gu et al., 2011; Gu and Wang, 2011; Wang et al., 2012; Gu, 2014; Li et al., 2021). Furthermore, molecular methods require sophisticated equipment and are not suitable for implementation in the field, causing delayed detection and response to PWD pandemics (Sultana et al., 2013). Therefore, a specific, sensitive, and field-applicable diagnostic method is needed to improve the efficiency of *B. xylophilus* detection and quarantine.

Alternatively, isothermal DNA amplification techniques that do not require the use of thermal cycling apparatus have been applied to detect *B. xylophilus*. These include loop-mediated isothermal amplification (LAMP) (Kikuchi et al., 2009; Meng et al., 2018; Ahuja and Somvanshi, 2021), denaturation bubble-mediated strand exchange amplification (SEA) (Liu C. et al., 2019), and recombinase polymerase amplification (RPA) (Cha et al., 2019, 2020; Fang et al., 2021). RPA, an isothermal nucleic acid amplification method that has attracted much attention because it is sensitive, rapid, works at isothermal temperature (Piepenburg et al., 2006; Glökler et al., 2021). In addition, RPA assays have been developed to detect some plant-parasitic and animal-parasitic nematodes, including *B. xylophilus* (Cha et al., 2019, 2020), *Meloidogyne enterolobii* (Subbotin, 2019), *Meloidogyne* spp. (*M. incognita*, *M. arenaria*, *M. javanica*, and *M. enterolobii*) (Ju et al., 2019), *Meloidogyne javanica* (Chi et al., 2020), *Meloidogyne hapla* (Song et al., 2020; Subbotin and Burbridge, 2021), *Angiostrongylus cantonensis* (Jarvi et al., 2021), and *Trichinella* spp. (Li et al., 2019).

Recombinase polymerase amplification amplicons can be visualized using lateral flow dipstick (LFD), with results could be directly interpreted by the naked eye within a few minutes (Daher et al., 2016). LFDs are products based on lateral flow technology using gold nanoparticles and can promptly detect amplification products making results easy to interpret (Qi et al., 2018).

**FIGURE 1:**
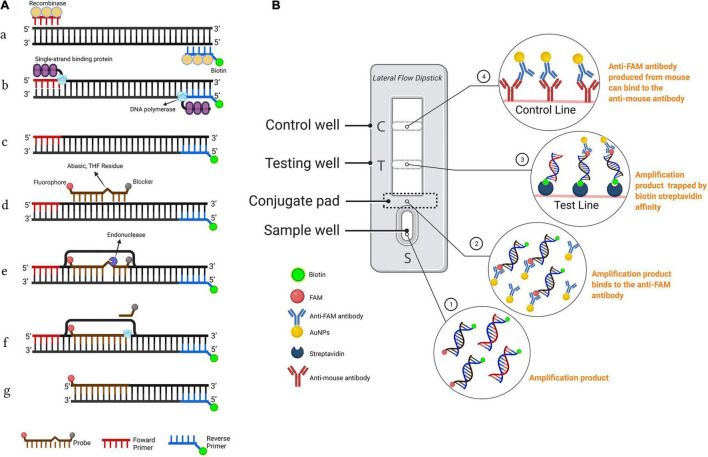
Schematic representation of the recombinase polymerase amplification combined with lateral flow dipstick (RPA-LFD) assay. **(A)** Principle of RPA. DNA strands are represented by horizontal lines, and base pairings are represented by short vertical lines between DNA strands. The forward primer (F), reverse primer (R), probe (P), nfo, single strand DNA-binding protein (SSB), polymerase, recombinase, fluorophore, blocker, and tetrahydrofuran (THF) residue are shown by various color and forms and text annotation. Created with BioRender.com. **(B)** Schematic representation of the lateral flow dipstick working principle. Lateral flow dipstick was painted after real ones. The segment names are displayed on the left of the dipstick drawing. The direction of liquid migration is from the sample well, through the conjugate pad, to the test and control wells. The coating material on each segment of the dipstick is shown in the image to the right. Molecules can be captured by the coating material on the test line and control line. Molecules and their symbolic shape are displayed in the lower-left corner. Dipstick created with BioRender.com.

The LFD assay requires a special nfo probe with a fluorescein amidites (FAM) (carboxyfluorescein) antigen label at the 5′-end, a tetrahydrofuran (THF) spacer (abasic site) in the middle, a C3 spacer (amplification blocker) at the 3′-end, and reverse primer labeled with biotin at the 5′-end. RPA amplicons are amplified using two primers and a probe, which contains both biotin and FAM, and the mouse anti-FAM antibody is enveloped with AuNPs. After reaction mixture is added to the sample well, they are driven by capillary force to move across the conjugate pad and bind to the anti-FAM AuNPs. The test line, which is enveloped with streptavidin, captures molecules with a biotin label when the amplification products go through. The control line, which is used to validate LFD detection, only captures the anti-FAM antibody enveloped with AuNPs because the anti-FAM antibody is from a mouse (Figure 1; Dai et al., 2019; Miao et al., 2019).

It should be noted that false-positive signals from the self-linked primer–probe complex should be considered a vital inherent defect of the RPA-LFD assay (Wu et al., 2020). Without the temperature cycles in PCR-based methods, primer combination in RPA is performed at ambient temperature, which does not provide an opportunity for dissociation if mispairing occurs and may generate a false-positive signal. In addition, LFD could not differentiate the size of the amplification product that releases signal. Furthermore, the RPA-LFD assay is overly sensitive; thus, interference from false-positive signals due to primer-dependent artifacts must be avoided (Poritz and Ririe, 2014; Wang et al., 2020).

In this study, we developed a simple, rapid, specific, and sensitive RPA-LFD assay to detect *B. xylophilus*. This method eliminated false-positive signals from non-specific primer–probe complexes due to an elaborate design and strict screening of primers and probe, as well as by bringing in base substitutions on the reverse primer and probe. The specificity and sensitivity of the assay were also investigated. The practicability was also analyzed by detecting *B. xylophilus* in spiked pine wood samples.

## Materials and Methods

### Specimen Collection and DNA Extraction

*Bursaphelenchus xylophilus*, *B. mucronatus*, and *Bursaphelenchus doui* were provided by the Key Laboratory of Forest Protection of National Forestry and Grassland Administration, Chinese Academy of Forestry, China. A total of 17 nematode isolates representing three *Bursaphelenchus* species, collected from multiple sites in China, were used in this study (Table 1). Nematode isolates were reared on *Botrytis cinerea* mycelia cultured on potato dextrose agar (PDA) plates at 25°C for 5–7 days and were identified using morphological methods and molecular diagnostic methods. Nematodes were then separated using the Baermann funnel technique (Viglierchio and Schmitt, 1983), and 500 μl of nematodes were collected in a 1.5 ml Eppendorf tube after being washed three times in sterilized water and stored at 4°C for future use.

**TABLE 1:** Nematode species used in *B. xylophilus* recombinase polymerase amplification combined with lateral flow dipstick (RPA-LFD) detection assay.

Species	Isolate	Host	Origin
*Bursaphelenchus xylophilus*	Bx-HS	*Pinus massoniana*	Huangshan, Jiangsu
	Bx-LY	*P. massoniana*	Liyang, Jiangsu
	Bx-CD9	*P. thunbergii*	Changdao, Yantai
	Bx-ZJ	*P. massoniana*	Fuyang, Zhejiang
	Bx-HZ	*P. massoniana*	Hangzhou, Zhejiang
	Bx-NB	*P. massoniana*	Ningbo, Zhejiang
	Bx-AMA3	*P. hwangshanensis*	Maanshan, Anhui
	Bx-YW4	*P. massoniana*	Kunming, Yunnan
*Bursaphelenchus mucronatus*	Bm-DB	*P. sylvestris* var. *mongolica*	Jiagedaqi, Heilongjiang
	Bm-NB	*P. massoniana*	Ningbo, Zhejiang
	Bm-BM7	*P. massoniana*	Wuhu, Anhui
	Bm-BM9	*P. massoniana*	Zigong, Sichuan
	Bm-JHL10	*P. elliottii*	Nanjing, Jiangsu
*Bursaphelenchus doui*	Bd-NJ	*P. massoniana*	Nanjing, Jiangsu
	Bd-CQ	*P. massoniana*	Yunyang, Chongqing
	Bd-ZG1	*P. elliottii*	Zigong, Sichuan
	Bd-ZG4	*P. elliottii*	Zigong, Sichuan

Genomic DNA (gDNA) was extracted from approximately 500 nematodes of each nematode species (*B. xylophilus*, *B. mucronatus*, and *B. doui*) using the TIANamp Genomic DNA Kit [Tiangen Biotech (Beijing) Co. Ltd., Beijing, China] according to the manufacturer’s instructions. The DNA concentration and purity were quantified using a NanoDrop2000 spectrophotometer (Thermo Fisher Scientific, Waltham, MA, United States). The DNA was diluted to 10 ng/μl with distilled water and stored at −20°C for future use.

### Designing of Recombinase Polymerase Amplification Primers

To design specific primers for RPA, the previously identified conserved gene encoding synaptic guidepost protein (SYG-2), was selected as the target segment (Gou, 2014; Meng et al., 2018). The syg-2 gene part sequences were amplified using PCR primer pairs syg2-part-f/syg2-part-r, and the PCR cycle conditions were set as described by Gou (2014). The PCR products (25 μl) were electrophoresed on a 2% agarose gel, and the amplified products were recovered using a PCR Cleaning Kit [Tiangen Biotech (Beijing) Co., Ltd., Beijing, China] and sequenced [Sangon Biotech (Shanghai) Co. Ltd., Shanghai, China], and the sequences were aligned using the ClustalW program implemented in MEGA version 7.0.14. RPA-basic assays require forward and reverse primers; accordingly, the RPA primers were designed using Primer Premier 5.0 (Premier Biosoft, Palo Alto, CA, United States) according to the TwistDx instruction manual (Cambridge, United Kingdom). RPA primer length was 30–36 bp, the primer GC content was 30–70%, and the amplicon length was between 150 and 300 bp.

### Recombinase Polymerase Amplification Procedure and Electrophoresis

The Basic RPA 50-μl reaction volume was prepared according to the instructions for the RPA-basic kit (#WLN8201KIT; AMP-Future Biotech Co. Ltd., Weifang, China). It consisted of 29.4 μl rehydration buffer, 2 μl forward primer, 2 μl reverse primer (primer concentration, 10 μM), one lyophilized enzyme pellet, 1 μl DNA template, and 13.1 μl nuclease-free water; the reaction was initiated by adding 2.5 μl magnesium acetate. After incubation at 38°C for 30 min, amplicons were purified using spin columns before electrophoresis on 1.5% agarose gels.

### Probe Designing

The probes were designed using Primer Premier 5.0 software (Premier Biosoft). The size of the probe was set as 46–53 nt, GC content was 20–80%, and T_m_ set as 50–80°C. Maximum hairpin and primer dimer parameters were set at ≤4 bonds near 3′-end. Other settings were set as default. Following design, primers and probes were assessed using the OligoAnalyzer Tool (IDT) and screened depending on their binding score for continuous base pairings between the probe and reverse primer. The RPA primers and nfo probe were synthesized and provided by Sangon Biotech (Shanghai) Co. Ltd.

### Recombinase Polymerase Amplification-Lateral Flow Dipstick Procedure

Recombinase polymerase amplification-lateral flow dipstick assays were carried out in a 50 μl reaction volume using the RPA nfo kit (#WLN8203KIT: AMP-Future Biotech Co. Ltd.) according to the manufacturer’s instructions. The reaction mixture comprised 29.4 μl rehydration buffer, 2.5 μl magnesium acetate, 2 μl each primer pair (10 μM), 0.6 μl nfo probe (10 μM), 12.5 μl nuclease-free water, and 1.0 μl DNA template. All reagents except the DNA template and magnesium acetate were prepared in a master mix, which was subsequently dispensed into reaction tubes containing a dried enzyme pellet provided with the kit. After adding 1 μl of the nucleic acid template to the tubes and pipetting 2.5 μl of magnesium acetate into the tube lids, the lids were closed carefully, and the tubes were inverted several times and briefly centrifuged at 6,000 rpm for 30 s.

Post-amplification results were visually interpreted using Hybridetect LFD (kit #WLFS8201; AMP-Future Biotech Co. Ltd.). Thereafter, 10 μl of reaction solution was added to a centrifuge tube containing 190 μl ddH_2_O, after which 50 μl diluted solution was pipetted onto the LFD sample well and incubated for 3–5 min at ambient temperature. If both test and control line are visible, it is positive; if only the control line is visible, it is negative; and if the control line is not visible, it is invalid. A repeat test would then be conducted using a new dipstick in an invalid situation.

### Introduction of Base Substitutions to Probe and Reverse Primer

Recombinase polymerase amplification can tolerate several base mispairings in primers without noticeably influencing accuracy (Daher et al., 2015; Liu X. et al., 2019). Therefore, base substitutions were introduced into the probe and reverse primer according to previous research (Wu et al., 2020). The probe and primer with base substitution were evaluated for false-positive signals using no template control (NTC).

### Optimization of Recombinase Polymerase Amplification-Lateral Flow Dipstick Assays

To investigate the optimal conditions of the RPA-LFD assay, different reaction temperatures (15, 20, 25, 30, 35, 40, and 45°C) and reaction times (5, 10, 15, 20, 25, 30, and 35 min) were assessed. For determination of optimal reaction temperature, all treatments were reacted for 30 min. After the reaction, tubes were immediately put on ice to stop the reaction.

### Specificity of Recombinase Polymerase Amplification-Lateral Flow Dipstick Assay

To investigate the specificity of the assay, nematode genomic DNA was prepared from 17 nematode isolates, including eight *B. xylophilus*, five *B. mucronatus*, and four *B. doui* isolates, were used as DNA templates, as described above. Specificity tests were repeated three times.

### Analysis of Recombinase Polymerase Amplification-Lateral Flow Dipstick Assay Sensitivity

The sensitivity of the RPA assay was investigated using the RPA nfo kit by testing a 10-fold serial dilution of gDNA extracted from *B. xylophilus*. Genomic DNA extracted from *B. xylophilus* was diluted into seven concentrations with sterile distilled water, including 10, 1, 10^–1^, 10^–2^, 10^–3^, 10^–4^, and 10^–5^ ng, respectively. Each dilution and sterile distilled water as the NTC, were used as templates in the RPA-LFD assay, respectively, as described previously. The sensitivity tests were repeated thrice.

### Preparation of Pinewood Spiked Sample for Recombinase Polymerase Amplification-Lateral Flow Dipstick Evaluation

To investigate the interference from the DAP buffer or pine wood, which includes humic acid, ethanol, polysaccharides, and polyphenols (Cha et al., 2019). Pine wood chips (100 mg) from healthy pine trees were placed in a 2 ml tube filled with 1 ml DAP buffer (20 mM sodium hydroxide, 5% polyethylene glycol 200, and 5% dimethyl sulfoxide) (Cha et al., 2019), and 10 ng of pure gDNA of different *B. xylophilus* or *Bursaphelenchus* spp. isolates was spiked into the lysates. The tubes were then vibrantly mixed and incubated at ambient temperature for 10 min. Subsequently, 2.5 μl of lysate solution was used as the RPA template (the final concentration of gDNA was 25 pg in each RPA reaction).

## Results

### Primer Design and Screening

**FIGURE 2:**
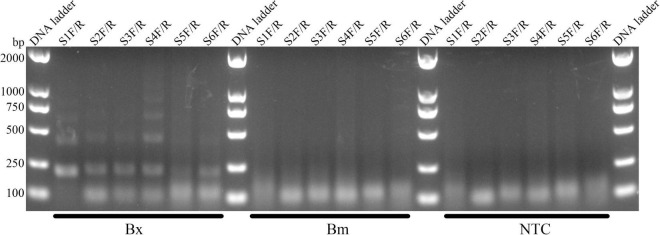
Screening of primer pairs by Basic-RPA. The image shows amplification bands targeting the syg-2 gene. The primer name is shown above each lane. The following Bx, Bm, and NTC lanes are *B. xylophilus*, *B. mucronatus* genomic DNA, and no template controls for corresponding primer pairs, respectively. Band sizes for DNA ladders are shown on the left.

Using Primer Premier 5.0, six potential primer pairs were obtained (Table 2). They were screened using basic RPA (Figure 2). The image showed amplification of the syg-2 (synaptic guidepost protein) target fragments, which have a typical ladder pattern, comprised multimers of the amplicon-sized monomer, probably because the protocadherin gene family which syg-2 belongs to is arrayed in tandem on the chromosome (Figure 2; Yamagata et al., 2003). However, five of them showed primer dimer bands under specific amplification bands. Primer pair S1F/R amplified specific products and showed no obvious primer-dimer (Figure 2). In order to use LFD to interpret results, the reverse primer was labeled at the 5′-end with biotin.

**TABLE 2:** Sequences of the primers and probes used in *B. xylophilus* recombinase polymerase amplification (RPA) assay.

Assay	Primer/Probe	Sequences 5′-3′	Length (bp)	Amplicon size (bp)
Basic RPA	S1F	TCTTACTGTCAGCAAATGA AATAATTAGGAGGAATC	36	194
	S1R	ATAGGCAGCAGAAGTTA GACAATCGGGAAT	30	
	S2F	TCTTACTGTCAGCAAATGA AATAATTAGGAGGAATC	36	208
	S2R	CGGAAAATACAAAAATA GGCAGCAGAAGTTAGA	33	
	S3F	TCTTACTGTCAGCAAATGA AATAATTAGGAGGAATC	36	209
	S3R	TCGGAAAATACAAAAATA GGCAGCAGAAGTT	31	
	S4F	ATTCTTACTGTCAGCAAAT GAAATAATTAGGAGGAA	36	210
	S4R	CGGAAAATACAAAAATAG GCAGCAGAAGTTAGA	33	
	S5F	ATTCTTACTGTCAGCAAA TGAAATAATTAGGAGGAA	36	211
	S5R	TCGGAAAATACAAAAATA GGCAGCAGAAGTT	31	
	S6F	CTGTCAGCAAATGAAATAA TTAGGAGGAATCCAATT	36	203
	S6R	CGGAAAATACAAAAATAG GCAGCAGAAGTTAGA	33	
RPA-LFD	S1R-nfo	[Biotin]ATAGGCAGCAGAAGT TAGACAATCGGGAAT	36	138
	S1-nfo-P	[FAM]GTATATTCATAATAGA GTTGTAAACACCGT[THF]TA AAGGAATTAGTTT[C3 Spacer]	45	
Modified RPA-LFD	mS1R-nfo	[Biotin]ATAGGCAGCAGAAG TAAGACAATCGGGAAT	36	138
	mS1-nfo-P	[FAM]GTATATTCATAATAGAG TTGTAAACACCGT[THF]GATA GGAATTAGTTT[C3 Spacer]	30	

### Addition of a Probe Into the Recombinase Polymerase Amplification Reaction

The nfo probe (S1-nfo-P) was between the forward and reverse primers and comprised of an oligonucleotide with a 5′-FAM as antigenic label and a 3′-C3 spacer that could block extension. An THF spacer (tetrahydrofuran) was placed on the probe to replace a guanine. The nfo enzyme activated after bases flanking THF matching with complementary bases and would cut THF site, removing probe 3′-end blocker for extension (Figure 1A). The specific RPA primers, nfo probe, and their sequences are listed in Table 2.

**FIGURE 3:**
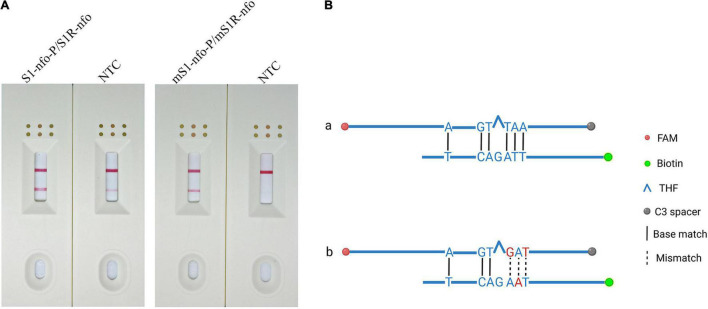
Preliminary test and analysis of the original primer–probe set and base-substituted primer–probe set. **(A)** The image shows the LFD results for RPA amplifications using original primer–probe set and base-substituted primer–probe set. The name of each set is displayed above dipstick. NTC is no template control tested for false-positive signals. The positions of test and control lines are marked on the right of the dipsticks image. The template was *B. xylophilus* gDNA. The reactions are carried out at 38°C for 10 min. **(B)** Analysis of the cross-dimer formation between the probe and reverse primer. The original probe has five-continuous base pairings with original reverse primer, which could lead to primer–probe complex, resulting in a false positive signal. After bringing in mispairings into the original self-ligating primer–probe set, one of which is on reverse primer and two on the probe, the formation of the probe-primer complex is artificially interrupted and signal is eliminated. Labels and modifications for DNA, nfo, base matches, and mismatches are represented by different shapes and colors, and a legend is given to the right of the figure. Created with BioRender.com.

For the newly added probe, the RPA-LFD assay was carried out to evaluate the amplification signal and false-positive signal. The amplification signal was evident for primer–probe set S1R-nfo/S1-nfo-P; however, the false-positive signal also existed for NTC (Figure 3A). Cross dimer analysis showed that there was still a continuous base match between the probe and primer. Furthermore, the matched bases were located on two sides of THF site, facilitating the nfo enzyme cutting activity (Figure 3B).

### Elimination of False-Positive Signals With Base Mismatches

The results showed that false-positive signal was eliminated by bringing mispairings on probe and reverse primer (mS1-nfo-P/mS1R-nfo) (Figures 3A,B, substituted bases in red). The sequences of the modified probe (mS1-nfo-P) and reverse primer (mS1R-nfo) are listed in Table 2. These base substitutions did not influence amplification accuracy and efficiency by observing band color density (Figure 3A). Therefore, this primer and probe were used for the following RPA-LFD reactions in this study.

### Optimization of Recombinase Polymerase Amplification-Lateral Flow Dipstick Assay for *Bursaphelenchus xylophilus* Detection

**FIGURE 4:**
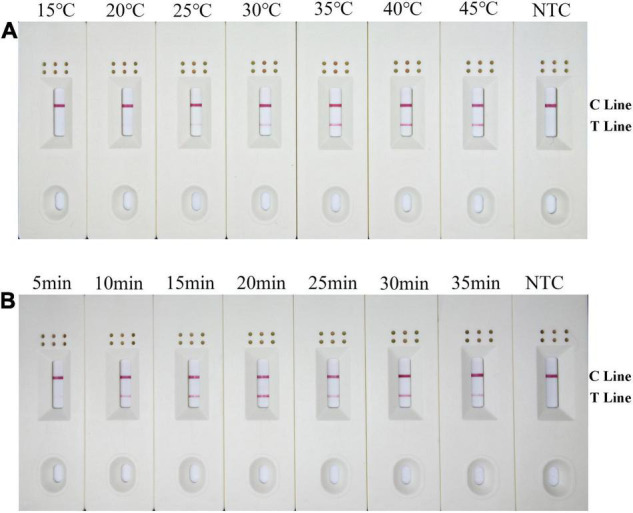
Optimization of the modified primer–probe set in RPA–LFD assay. **(A)** Optimal reaction temperature of the RPA–LFD assay. The figure shows the LFD results of RPA amplification at different temperatures. The temperature is displayed at the top of each dipstick. The amplification template is *B. xylophilus* gDNA. The NTC band is no template control carried out at 38°C. The positions of the control and test lines are shown on the right side of the dipstick. **(B)** Optimal reaction time of the RPA–LFD assay. The image shows the LFD results of RPA amplifications with different times. The time to perform the RPA reaction is displayed at the top of each dipstick. The amplification template is *B. xylophilus* gDNA. NTC dipsticks are performed for 10 min without template. The positions of the control and test lines are shown on the right side of the dipstick.

The RPA-LFD assays were tested at temperature range from 15 to 45°C at 5°C intervals for 30 min. Results showed that test lines were visible from 20 to 45°C, color density of the test line did not increase noticeably from 35 to 45°C. Therefore, 38°C was selected as the optimal temperature (Figure 4A). Moreover, the RPA-LFD reaction time was tested from 5 to 35 min at 5 min intervals at 38°C. Results showed that test lines were visible when the reaction time was 10–35 min; therefore, 10 min was selected as the optimal time for the RPA-LFD assay of *B. xylophilus* detection considering rapid diagnostics (Figure 4B).

### Analytical Specificity of Recombinase Polymerase Amplification-Lateral Flow Dipstick Assay

**FIGURE 5:**
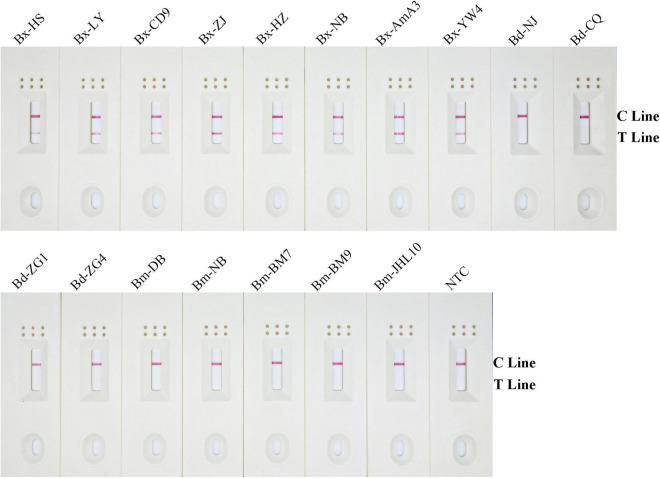
Detection specificity of the modified primer–probe set in RPA–LFD assay. Specificity determination of RPA-LFD using different nematode isolates gDNA templates. The isolate of nematode is marked above each dipstick. The NTC dipstick is no template control. The positions of test and control lines are shown on the right side of dipstick. Reactions are carried out at 38°C for 10 min.

To assess the RPA-LFD analytical specificity, gDNA was prepared using several nematode species, including eight *B. xylophilus*, five *B. mucronatus*, and four *B. doui* isolates (Table 1). Only when *B. xylophilus* gDNA as DNA template can produce red test lines on dipsticks, indicating positive results. When other nematode species gDNA or NTC as DNA template, no red test lines were seen on dipsticks (Figure 5), indicating that this primer–probe combination had good specificity toward *B. xylophilus* and had no cross-reactions with other nematode species.

### Detection Limit of Recombinase Polymerase Amplification-Lateral Flow Dipstick Assay

**FIGURE 6:**
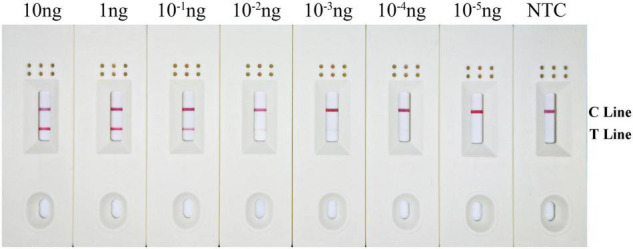
Detection limit of the RPA–LFD assay. The image shows the LFD results of RPA amplification with different concentrations of *B. xylophilus* gDNA. The DNA concentration used as template is shown above each dipstick. NTC is the no template control. The reactions are carried out at 38°C for 10 min. Test line and control lines are shown on the right side of dipstick.

Results showed that the LFD test and control lines were displayed from 10 to 10^–3^ ng gDNA. Furthermore, the red color of the test line lightened with decreasing *B. xylophilus* concentrations, and *B. xylophilus* gDNA as low as 1 pg (10^–3^ ng) could be detected (Figure 6). Therefore, the detection limit of the RPA-LFD assay for *B. xylophilus* was 1 pg of gDNA.

### Application Simulation of the Recombinase Polymerase Amplification-Lateral Flow Dipstick Test for *Bursaphelenchus xylophilus* Detection

**FIGURE 7:**
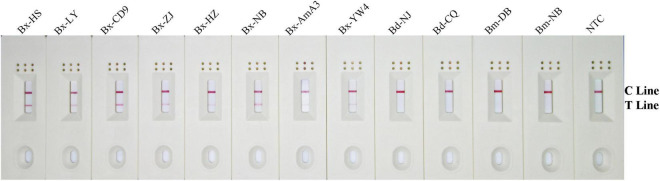
Detection of *B. xylophilus* purified gDNA in spiked pinewood samples. Images of the LFD results of RPA amplification of healthy pinewood samples spiked with gDNA of various nematode isolates. The spiked gDNA species are shown at the top of each dipstick. The double-distilled water was used as the no-template control, corresponding to the NTC dipstick. The positions of the control and test lines are shown on the right side of the image. The reactions are carried out at 38°C for 10 min.

Using an application simulation, the RPA-LFD test was applied for *B. xylophilus* detection using artificially spiked pine wood samples. Thirteen healthy pine wood samples were prepared, of which eight were artificially spiked with *B. xylophilus* gDNA, two with *B. mucronatus* gDNA, another two with *B. doui* gDNA, and one with sterilized distilled water as NTC. The results of the RPA-LFD assay were consistent with those of the spiked gDNA species and all eight *B. xylophilus* gDNA spiked pine wood samples were successfully detected (Figure 7).

## Discussion

*Bursaphelenchus xylophilus* induced PWD has destroyed numerous pine trees and has spread to 18 provinces and 693 counties in China over the past 40 years, causing considerable economic losses of hundreds of billions of yuan [Announcement of the State Forestry and Grassland Administration (No. 6 of 2021), 2021]. Therefore, a primary procedure for disease prevention and quarantine comprising an accurate, rapid, sensitive, and convenient method for detecting *B. xylophilus* in pine wood is urgently needed.

Polymerase chain reaction-based amplification is considered the gold standard of molecular diagnostics; however, it requires trained personnel and specialized, expensive equipment generally unavailable outside laboratory settings (Patel et al., 2014). As an emerging isothermal amplification technique, RPA has displayed many strengths, such as short detection time, high sensitivity, easy operation, and minimum instrument needed. Besides, the RPA amplification results could be interpreted using LFD within a few mins. All these characteristics make RPA-LFD very suitable for on-site detection (James and Macdonald, 2015; Li et al., 2019).

The choice of detection target is crucial for specific diagnostics. Several genes have been selected as biomarkers for PWN detection, including 5S rRNA, hsp70, satellite DNA, ITS rRNA, topoisomerase I, syg-2, and pel-3 (Kang et al., 2004; Castagnone et al., 2005; Leal et al., 2007; Huang et al., 2010; Zhuo et al., 2011; Kang et al., 2015; Meng et al., 2018). Among these, the syg-2 gene has been reported to have superior performance in the LAMP method and was thus selected as the target in this study. To identify regions specific to *B. xylophilus*, the syg-2 gene sequence of *B. xylophilus* was amplified using the PCR primers designed by Gou (2014) and then compared with two closely related species, after which the diverged region specific to *B. xylophilus* was selected (Supplementary Figure 1). The diverged region was considered a possible target area for designing primer and probe in the assay. Since the protocadherin DNA family has been shown to consist of tandemly arranged repeats, the amplification of a ladder of multimers of the amplicon-sized monomer was observed in basic-RPA experiments using the designed primers (Figure 2; Hirano et al., 2003; Washbourne et al., 2004).

On the LFD, the control line is enveloped with anti-mouse antibody, and the test line was enveloped with streptavidin. The test line first captures products labeled with biotin when the reaction mixture passes through. If the products also had a FAM label, it will combine with the AuNPs, which are enveloped with anti-FAM antibody, led to the enrichment of AuNPs at the test line, showing a red line (Wang et al., 2020). The control line is used to validate LFD detection, only captures the anti-FAM antibody enveloped with AuNPs, because the anti-FAM antibody is from a mouse (Figure 1).

**FIGURE 8:**
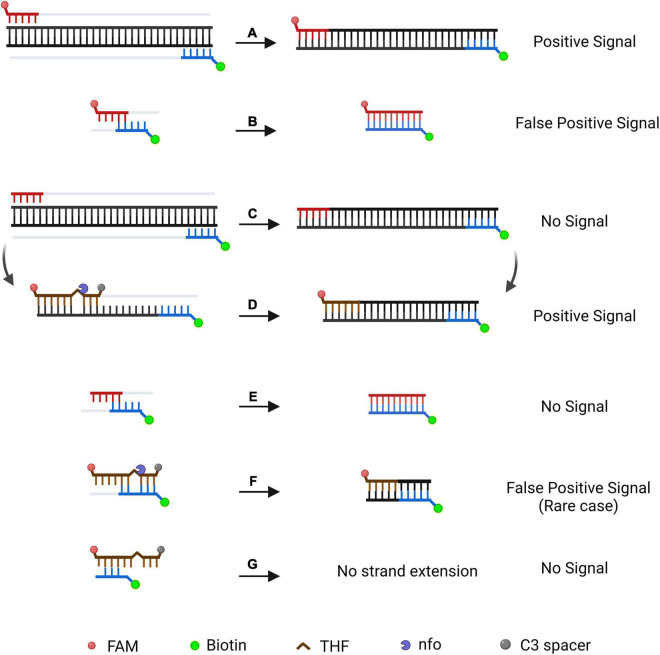
Schematic illustration of a specially designed probe that reduces false-positive signals from primer-dependent artifacts. In the RPA-Basic-based LFD reaction, both the amplification products **(A)** and the primer–dimers **(B)** can give positive signals. By using a probe in RPA reaction, amplification of the target DNA from the primers does not yield a positive signal **(C)**. The amplification product is subjected to another round of amplification under the guidance of the probe and shows a positive signal **(D)**. Primer dimers in the probe-based RPA reaction give no positive signal **(E)**. In most cases, the primer–probe complex will not give a positive signal **(G)**. The complex gives a positive signal when the primer and probe pairing multiple bases flanking the THF site **(F)**. DNA strands are represented by horizontal lines, and matching bases are represented by short vertical lines between the DNA strands. The expected amplification of the DNA strand is indicated by the gray line. Modification group and nfo are represented by different colors and forms, and a legend is given at the bottom of the figure. Created with BioRender.com. Picture inspired by Wu et al. (2020).

Nonetheless, the RPA-LFD method has a non-ignorable inherent defect of primer-dependent artifacts. In the RPA-basic-based LFD reaction, forward and reverse primers were labeled with FAM and biotin at 5′-end, respectively. Amplicons are labeled with both biotin and FAM, if primer dimers are formed, it also produces a positive signal (Figures 8A,B). No matter how cautious the screening of primer is, it is nearly impossible to prevent primer dimers in the DNA amplification process (Meagher et al., 2018). Thermal cycling strategies could prevent primer-dimer formation in PCR but are unsuitable for RPA because RPA works at an isothermal temperature of about 38°C, and mispaired primers are difficult to dissociate (Brownie et al., 1997). Furthermore, the LFD method cannot differentiate the size of the molecule emitting signal and treats each molecule as a positive signal (Safenkova et al., 2020). Considering the RPA-LFD method is highly sensitive (Miao et al., 2019), any interference from primer-dependent artifacts could result in false-positive detection.

Research has shown that introducing a probe in RPA would enhance specificity and decrease non-specific amplification products (Figures 8C,D; Piepenburg et al., 2006; Ivanov et al., 2021). FAM was labeled at the 5′-end of the probe, while the C3-spacer was labeled at the 3′-end of the probe that block extension. The THF site was placed on the probe to be cut by nfo enzyme. In this way, amplification led by primer pair would result in a product that only has biotin label, which would not generate a signal on the LFD (Figure 8C). The probe fully pairing amplified DNA strand can be cut by nfo at THF site (nfo was activated if bases flanking THF site paired with complementary bases), freeing the 3′-end for the extension to produce a positive signal on the LFD (Figure 8D). Using this probe-guided amplification, primer-dimers did not produce a signal (Figure 8E). The partly matched primer–probe complex could not be amplified (Figure 8G). Only in the rare case, the primer and probe paired several bases flanking the THF site (Figure 8F), and this would generate false-positive signal (Wu et al., 2020).

Therefore, elimination of the false-positive signal could not only count on using a probe, elaborate design and strict screening are still essential to prevent false-positive scenarios (Figure 8). Based on previous research, we inferred that the false-positive signal occurred in this study should be caused by the probe-primer complex since the labels were on the probe and reverse primer (Wu et al., 2020). By setting stringent parameters, probes were designed to have the lowest theoretical chance of primer pairing. However, cross-dimer analysis displayed many possible primer–probe dimer scenarios between the nfo probe and the reverse primer, and some cross-dimers fit the special case of false-positive signals. That is, the reverse primer formed mismatches with bases flanking the THF site on the probe, which could elucidate why the original primer–probe set (S1-nfo-P/S1R-nfo) displayed false-positive signals.

The probe needed to be in the middle of two primers. However, obtaining an ideal probe without any continuous bases pairing to the reverse primer was scarcely possible. Nonetheless, we could exploit the tolerance of RPA to certain base mispairings on primers against the template, and attempt to bring in base substitutions in RPA-LFD method (Daher et al., 2015; Liu X. et al., 2019). By imitating a previously described method of bringing in three mismatches (Wu et al., 2020) in which two are in the probe and one in the reverse primer, the probe-primer dimer ligation was artificially interrupted, and false-positive was avoided. In addition, the RPA-LFD efficacy was not noticeably influenced when compared the color difference between the S1-nfo-P/S1R-nfo line and mS1-nfo-P/mS1R-nfo line, as shown in Figure 3.

Primer-dependent artifacts were successfully eliminated, resulting in the establishment of the RPA-LFD method. The analytical specificity test showed that the assay displayed high specificity toward *B. xylophilus* and avoided cross-reactions with the other two *Bursaphelenchus* species tested. A previous study reported that the detection limit of the RPA assay is approximately 1.6 fg (1.6 × 10^–6^ ng) of gDNA from *B. xylophilus* (using pure gDNA) (Cha et al., 2020). However, in our RPA-LFD assay, a sensitivity test using *B. xylophilus* gDNA showed that the detection limit was 1 pg. This is probably because the RPA probe-based approach had a lower sensitivity than the approach with labeled primers (Ivanov et al., 2021).

**FIGURE 9:**
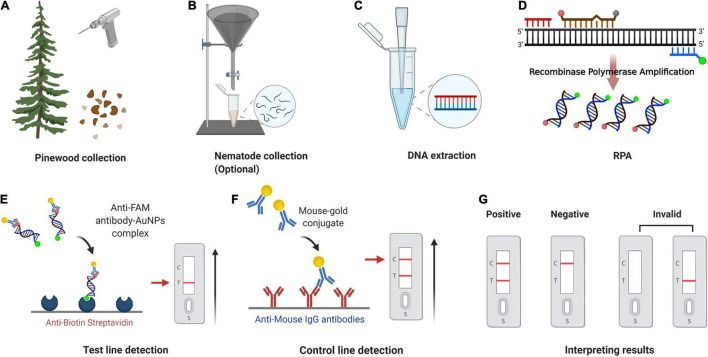
Point-of-Care testing workflow for pinewood nematode detection. **(A)** Pinewood chips are collected from trees using an electric drill or axe. **(B)** Nematodes are collected from pinewood chips using Baermann funnel method (optional). **(C)** gDNA is extracted from nematode/pinewood using DAP lysis buffer. **(D)** Recombinase polymerase amplification. **(E)** The sample enters testing well (T) and anti-FAM antibody-AuNPs complex binds to immobilized anti-biotin streptavidin. **(F)** Mouse antibody-gold conjugate binds to immobilized anti-mouse IgG antibodies. **(G)** Positive result: one dipstick each in C line and T line; Negative result: one dipstick in C line; Invalid result: no dipstick in C line despite the T line. The whole procedure for the RPA-LFD assay could be finished under 38°C within about 30 min, including 15 min for nematode gDNA extraction and master mix preparation, 15 min for the RPA-LFD assay (10 min for RPA reaction, 5 min for visual detection on the LFDs). Adapted from “COVID-19 Serologic Diagnostic Test through Antibody Detection,” by BioRender.com (2020). Retrieved from https://app.biorender.com/biorender-templates.

The whole procedure for the RPA-LFD assay could be finished under 38°C within about 30 min, including 15 min for nematode gDNA extraction and master mix preparation, 15 min for the RPA-LFD assay (10 min for RPA reaction, 5 min for visual detection on the LFDs) (Figure 9). Compared with other diagnostic methods for *B. xylophilus* detection, the time needed for RPA-LFD detection was the shortest. Furthermore, RPA displayed tolerance to interference from pinewood (Figure 7), presenting an opportunity for further investigation into the prevalence of *B. xylophilus*, especially in new areas of occurrence.

However, applying RPA-LFD detection to PWN diagnostics still faces some issues, with the expense being a major one. Reagent costs for RPA-LFD assays are approximately 50 RMB per reaction at present, even when using the RPA nfo kit manufactured locally, which are higher than those for polymerase chain reaction. However, when considering instrumentation costs, the RPA-LFD method becomes relatively more affordable. Even though PCR is currently considered the gold standard for nematode diagnostics, thermocycling instruments are expensive. Local forestry bureaus or quarantine departments are not commonly equipped with these, results usually take days to weeks to obtain due to the shortage of instruments. The minimal equipment needed for conducting RPA-LFD assays would allow reactions to be completed timely in the field or at quarantine departments for point-of-care diagnostics within minutes rather than days.

The relatively low cost of RPA-LFD assay can be used by more people to support extensive surveillance across the country, whether in areas known to have high levels of PWD infection, such as Zhejiang Province, or in areas with relatively low levels of infection regions, supporting decision-making. In addition, detection capability in remote areas with limited resources could drastically increase with the emergency deployment of RPA-LFD assay kits. Thus, the RPA-LFD assay developed here has potential applications in the field and areas with limited resources.

## Conclusion

In conclusion, a rapid, on-site RPA-LFD assay for specific and sensitive detection of *B. xylophilus* was developed. The RPA-LFD assay prevents the risk of false-positive results from primer-dependent artifacts by using a probe and bringing base substitutions in the primer and probe. Furthermore, the assay successfully detected *B. xylophilus* with high specificity and sensitivity in less than 30 min at an isothermal temperature of 38°C. The developed RPA-LFD assay can provide a novel alternative for PWN point-of-care testing that has a simple read-out system and can be performed under field conditions without any special instrumentation or in areas with minimal laboratory infrastructure.

## Data Availability Statement

The original contributions presented in the study are included in the article/Supplementary Material, further inquiries can be directed to the corresponding author.

## Author Contributions

QZ conceptualized and designed the research, analyzed the data, interpreted the results, and wrote the manuscript. QZ and YL performed the experiments. QL, YL, ZW, and HW participated in the discussion for experimental design. XZ helped funding acquisition. QL revised the manuscript and directed the project. All authors contributed to the article and approved the submitted version.

## Conflict of Interest

The authors declare that the research was conducted in the absence of any commercial or financial relationships that could be construed as a potential conflict of interest.

## Publisher’s Note

All claims expressed in this article are solely those of the authors and do not necessarily represent those of their affiliated organizations, or those of the publisher, the editors and the reviewers. Any product that may be evaluated in this article, or claim that may be made by its manufacturer, is not guaranteed or endorsed by the publisher.

## Supplementary Material

Supplementary Figure 1 Nucleotide sequence alignment of the syg-2 gene part sequence from *Bursaphelenchus xylophilus* (Bx), *Bursaphelenchus mucronatus* (Bm), and *Bursaphelenchus doui* (Bd). Location of S1F/R primers and S1-nfo-P are marked by black arrow lines, respectively. Gaps in sequence alignment were indicated by dots.
