# Supplementary figures and images for: Rapid On-Site Detection of the Bursaphelenchus xylophilus Using Recombinase Polymerase Amplification Combined With Lateral Flow Dipstick That Eliminates Interference From Primer-Dependent Artifacts
Source: Front Plant Sci. 2022 Mar 18;13:856109. doi: 10.3389/fpls.2022.856109 (PMC8971978; doi:10.3389/fpls.2022.856109)

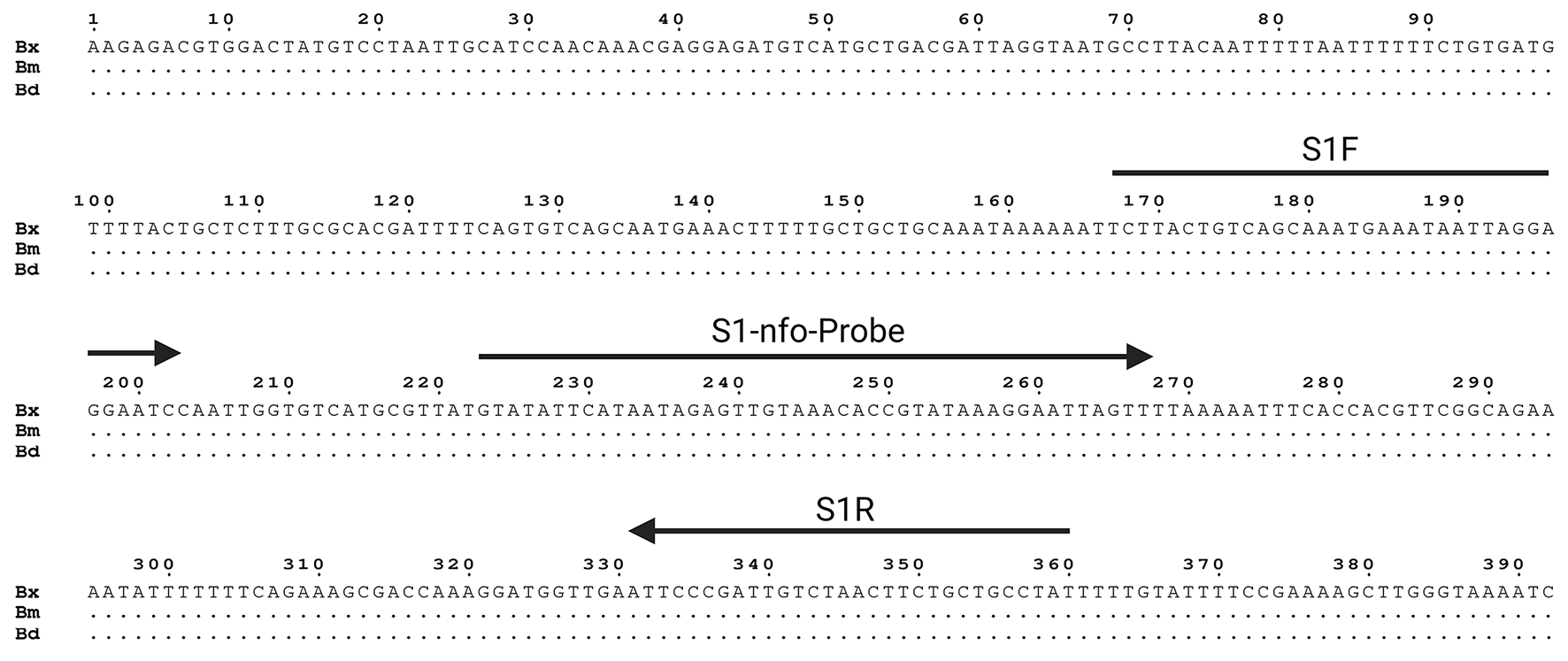

Supplement: Supplementary Figure 1 — Nucleotide sequence alignment of the syg-2 gene part sequence from Bursaphelenchus xylophilus (Bx), Bursaphelenchus mucronatus (Bm), and Bursaphelenchus doui (Bd). Location of S1F/R primers and S1-nfo-P are marked by black arrow lines, respectively. Gaps in sequence alignment were indicated by dots. [file Image_1.TIF]
